# Supplementary material for: Complex‐centric proteome profiling by SEC‐SWATH‐MS
Source: Mol Syst Biol. 2019 Jan 14;15(1):e8438. doi: 10.15252/msb.20188438 (PMC6346213; doi:10.15252/msb.20188438)
Supplement: Supplementary file 7 — Dataset EV6 [file MSB-15-e8438-s007.zip › feature_plots_bioplex/Q8N5Y8.pdf]

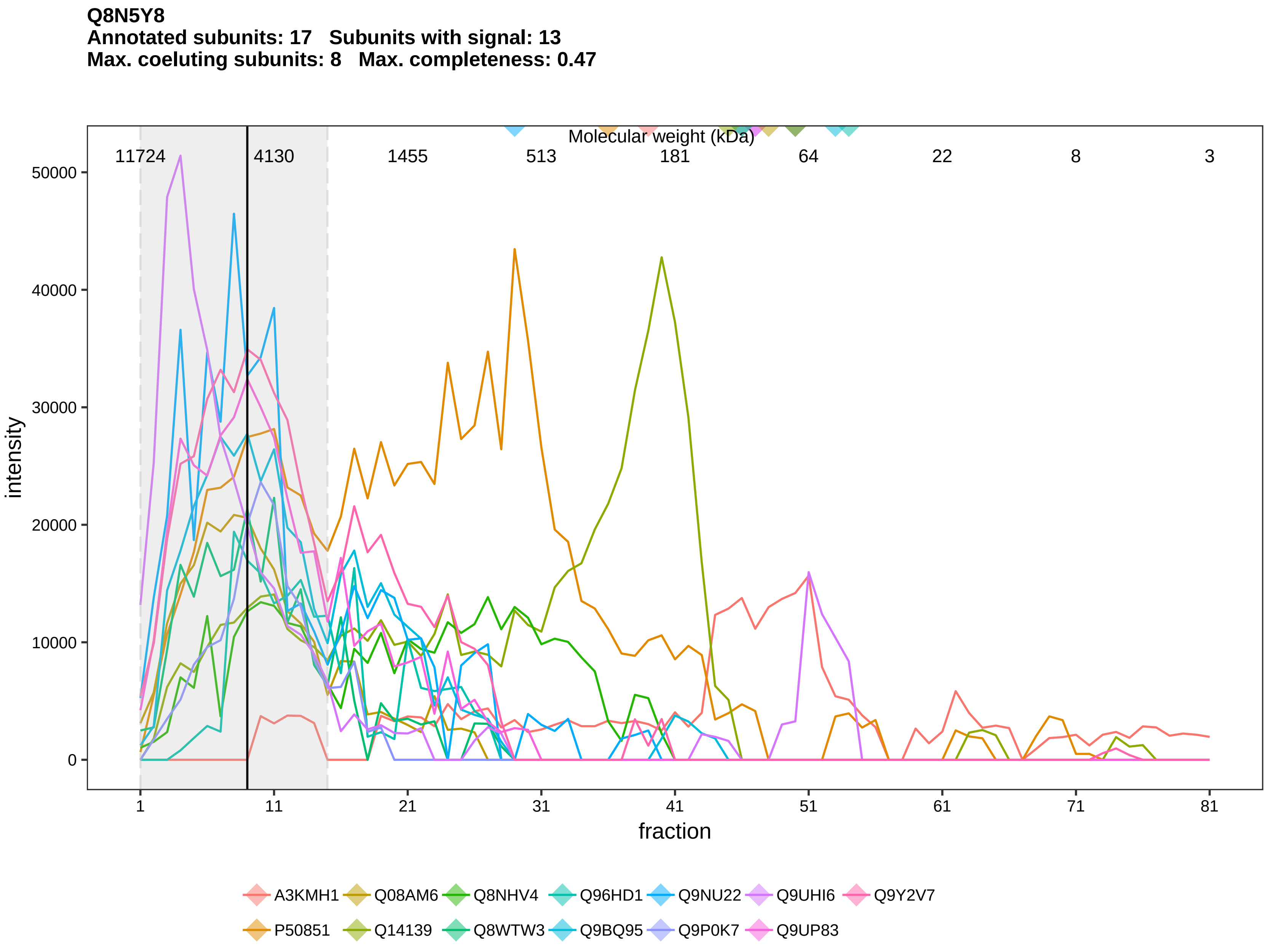

Q8N5Y8
Annotated subunits: 17 Subunits with signal: 13
Max. coeluting subunits: 8 Max. completeness: 0.47
Molecular weight (kDa)
11724
4130
1455
513
181
64
22
8
3
50000
40000
30000
20000
10000
0
intensity
1
11
21
31
41
51
61
71
81
fraction
A3KMH1
Q08AM6
Q8NHV4
Q96HD1
Q9NU22
Q9UHI6
Q9Y2V7
P50851
Q14139
Q8WTW3
Q9BQ95
Q9P0K7
Q9UP83
